# 25-hydroxyvitamin D level is associated with greater grip strength across adult life span: a population-based cohort study

**DOI:** 10.1530/EC-22-0501

**Published:** 2023-03-28

**Authors:** Fabienne A U Fox, Lennart Koch, Monique M B Breteler, N Ahmad Aziz

**Affiliations:** 1 Population Health Sciences, German Center for Neurodegenerative Diseases (DZNE), Bonn, Germany; 2 University for Health Sciences, Medical Informatics and Technology (UMIT TIROL), Tirol, Austria; 3 Institute for Medical Biometry, Informatics and Epidemiology (IMBIE), Faculty of Medicine, University of Bonn, Bonn, Germany; 4 Department of Neurology, Faculty of Medicine, University of Bonn, Bonn, Germany

**Keywords:** vitamin D, grip strength, sarcopenia, muscle strength, cohort study, ageing

## Abstract

**Objective:**

Maintaining muscle function throughout life is critical for healthy ageing. Although *in vitro* studies consistently indicate beneficial effects of 25-hydroxyvitamin D (25-OHD) on muscle function, findings from population-based studies remain inconclusive. We therefore aimed to examine the association between 25-OHD concentration and handgrip strength across a wide age range and assess potential modifying effects of age, sex and season.

**Methods:**

We analysed cross-sectional baseline data of 2576 eligible participants out of the first 3000 participants (recruited from March 2016 to March 2019) of the Rhineland Study, a community-based cohort study in Bonn, Germany. Multivariate linear regression models were used to assess the relation between 25-OHD levels and grip strength while adjusting for age, sex, education, smoking, season, body mass index, physical activity levels, osteoporosis and vitamin D supplementation.

**Results:**

Compared to participants with deficient 25-OHD levels (<30 nmol/L), grip strength was higher in those with inadequate (30 to <50 nmol/L) and adequate (≥50 to ≤125 nmol/L) levels (*ß*_inadequate_ = 1.222, 95% CI: 0.377; 2.067, *P* = 0.005; *ß*_adequate_ = 1.228, 95% CI: 0.437; 2.019, *P* = 0.002). Modelling on a continuous scale revealed grip strength to increase with higher 25-OHD levels up to ~100 nmol/L, after which the direction reversed (*ß*_linear_ = 0.505, 95% CI: 0.179; 0.830, *P* = 0.002; *ß*_quadratic_ = –0.153, 95% CI: –0.269; -0.038, *P* = 0.009). Older adults showed weaker effects of 25-OHD levels on grip strength than younger adults (*ß*_25OHDxAge_ = –0.309, 95% CI: –0.594; –0.024, *P* = 0.033).

**Conclusions:**

Our findings highlight the importance of sufficient 25-OHD levels for optimal muscle function across the adult life span. However, vitamin D supplementation should be closely monitored to avoid detrimental effects.

## Introduction

Maintaining muscle function throughout life is critical for healthy ageing (1). Progressive loss of muscle mass and function with age is a feature of primary sarcopenia and negatively affects mobility, functional independence and quality of life (2). It also increases the risk of falls and leads to higher healthcare costs and mortality risk (3, 4, 5). Primary sarcopenia has been estimated to affect between 6% and 22% of older adults, making it a major public health burden (5). Its onset may be as early as young adulthood (6). One of the diagnostic criteria of sarcopenia is decreased handgrip strength, a reliable proxy of overall muscle function, which can be measured easily and objectively (4, 5).

Both human and animal *in vitro* studies have shown that vitamin D can modulate skeletal muscle cell function (7). Muscle tissue expresses vitamin D receptors, and two biological pathways have been identified through which 1,25-dihydroxyvitamin D (1,25(OH)_2_D_3_), the biologically active form of vitamin D, could act on muscle tissue. The first pathway activates gene transcription and subsequent protein synthesis, which improves muscle function and structure and promotes cell differentiation and proliferation of type 2 muscle fibres (7, 8, 9). The second pathway has been hypothesized to rely on second-messenger pathways and membrane receptors, which are activated by 1,25(OH)_2_D_3_. This could lead to rapid calcium influx and uptake and affect muscle contraction (7, 9, 10). Given the high prevalence of vitamin D deficiency (11), optimizing vitamin D levels may be an easily actionable and cost-effective preventive and curative approach against sarcopenia and age-associated decline of muscle strength.

Although *in vitro* experiments indicate a causal relationship between low vitamin D levels and decreased muscle function (7, 8, 10), findings from observational studies have been inconsistent (12, 13, 14, 15, 16). While a previous cohort study found higher vitamin D levels to be related to greater upper arm strength, but not grip strength (13), other studies observed positive effects on grip strength, although exclusively in men (15, 17), women (18) and/or older adults (15, 19, 20, 21). Discrepancies in previous findings may be attributed to small sample sizes, usually restricted to a specific age or patient group, ethnicity or sex and residual confounding due to, for example, lack of information on season of measurement and vitamin D supplementation.

While the association between vitamin D levels and grip strength in adults above 65 years of age and athletes has been extensively studied (12, 22), few studies have assessed this relation in young- and middle-aged adults. The aim of this study was, therefore, to examine the relation between serum 25-hydroxyvitamin D (25-OHD) concentration and handgrip strength in adults across a wide age range in a population-based cohort study. In addition, we aimed to assess whether the association differed between men and women across age and between seasons.

## Methods

### Study participants

This study used cross-sectional baseline data from the first 3000 participants (age range: 30–95 years (*n* = 50–95 years) of the Rhineland Study, an ongoing population-based cohort study in Bonn, Germany (23). Participants were recruited from March 2016 to March 2019. They are required to be at least 30 years old and to have a sufficient command of the German language to provide informed consent. Participants complete comprehensive health assessments, including anthropometric and cardiovascular measurements, structured interviews as well as physical activity and fitness recordings. No financial incentives were offered for study participation. The study was carried out in accordance with the principles of the Declaration of Helsinki and was approved by the Medical Ethics Committee of the University of Bonn.

**Figure 1:**
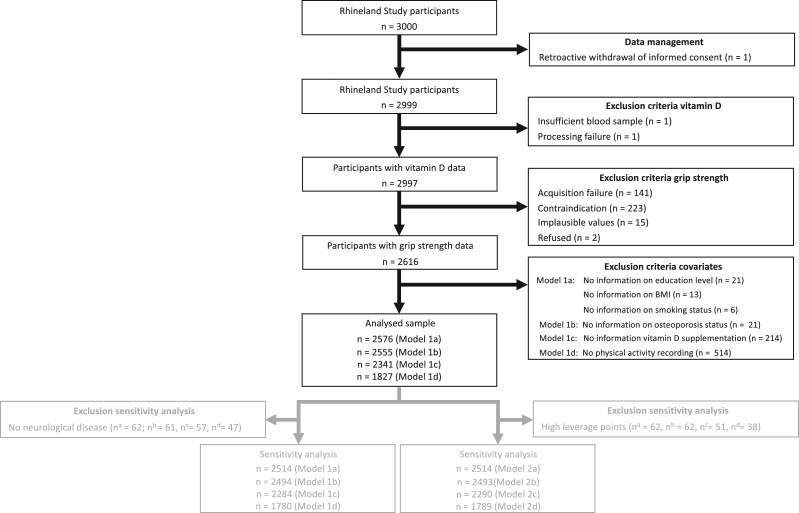
Recruitment flowchart.

Our analytical sample consisted of 2576 participants out of the first 3000 participants (Fig. 1). Serum 25-OHD data of two participants was missing due to acquisition and processing failures, while 381 participants had no grip strength data due to the following reasons: refusal to participate (*n* = 2), technical/acquisition failure (*n* = 141) or ineligibility (*n* = 223). Participants were deemed ineligible if they had an amputation or a fracture of the tested arm within the last month. They were also excluded from participation if they were currently suffering from pain in the tested limb or if pain could be induced by exerting force. In addition, we excluded 15 participants with implausible, and therefore likely erroneous, grip strength values. Lastly, we excluded participants with missing covariate data: for model 1a, we excluded 40 participants with missing body mass index (BMI) and education and smoking data; for model 1b, we additionally excluded 21 participants with missing osteoporosis status; for model 1c, we excluded 235 participants with missing vitamin D supplementation data and for model 1d, we excluded 514 participants with a missing or invalid accelerometer recording.

### Vitamin D measurements

Venous blood was withdrawn after overnight fasting. Blood samples were collected in tubes (S-Monovetten 7.5 mL tubes with coagulation) and kept at room temperature for 30 min for clotting to occur, followed by centrifugation for 15 min at 2000 ***g*** at 4ºC. Samples were automatically divided into aliquots (500 µL each) in FluidX 0.7 mL tubes and frozen at –80°C. Serum 25-OHD concentrations were assessed using the non-competitive chemiluminescent enzyme immunoassay (Lumipulse, FujiRebio, Ghent, Belgium), as described previously (24). For this assay, the lower limit of detection was 10 nmol/L. To account for values below the detection threshold (1.7% of observations), we set those values to a constant of 7, which was computed by dividing the lower limit of detection by the square root of two (25).

### Handgrip strength assessment

Handgrip strength was measured using the handheld Jamar Plus Digital Dynamometer (Patterson Medical, Patterson, MD, USA) according to American Society of Hand Therapists (ASHT) and Southampton protocol recommendations (26, 27). Participants were instructed to refrain from intensive exercise for 12 h prior to the assessment. To determine hand dominance, participants were asked with which hand they cut paper or bread. Previous studies reported a substantially higher maximum grip strength in the dominant hand compared to the nondominant hand (28). Therefore, for ambidextrous participants (*n* = 71, 2.8%), we defined the dominant hand based on the maximum measured grip strength across both hands. The maximum grip strength of the dominant hand was based on the measured grip strength of each hand across three trials. Starting with the right hand, the grip strength of each hand was recorded intermittently. The examination was carried out in a neutral sitting position, and participants were asked to continuously increase the applied force to the maximum on command.

### Assessment of covariates

Participants’ highest education level was determined using the International Standard Classification of Education 2011 (ISCED 2011) and was coded as low (lower secondary education or below), middle (upper secondary education to undergraduate university level) or high (postgraduate university study). During a medical interview, a medical history was obtained, including whether the participants had a history of osteoporosis, dementia, multiple sclerosis, Parkinson’s disease and stroke. We recoded osteoporosis status and neurological diseases into a binary variable (i.e. ‘yes’/’no’). Age, sex and smoking status were determined via self-report. We imputed missing smoking values based on cotinine metabolite levels. Participants were classified as current smokers if they had a cotinine level exceeding the non-smoker sample-defined 97.5 percentile. Vitamin D supplementation status was based on whether participants had taken vitamin D supplements for at least 30 days in the last 12 months or whether they regularly took prescribed cholecalciferol (ATC A11CC05). Height and weight were measured with a wireless measuring station (SECA 285). Physical activity was continuously recorded with activPAL3 accelerometers across 7 days. We calculated average daily energy expenditure (in metabolic equivalent hours) weighted by sensor wear time, as described previously (23).

### Statistical analysis

Sample characteristics are summarized using mean and s.d. for continuous variables and number and percentages for categorical variables. Differences between included and excluded participants were examined using binomial logistic regression while adjusting for age and sex.

We used multivariate linear regression models to assess the relation between continuous and categorical circulating 25-OHD levels (independent variable) and maximum handgrip strength of the dominant hand (outcome). We categorized 25-OHD levels using the Endocrine Society (deficient (<50 nmol/L), insufficient (≥50 to <75 nmol/L) and sufficient (≥ 75 nmol/L)) and the National Academy of Medicine (NAM) and National Institutes of Health (NIH) guidelines (deficient (<30 nmol/L), inadequate (30 to <50 nmol/L), adequate (≥50 to ≤125 nmol/L) and potential adverse (>125 nmol/L)). To test for potential nonlinear effects of vitamin D levels on grip strength, we included a quadratic term for 25-OHD in our models examining the effects of continuous circulating 25-OHD levels. The quadratic 25-OHD term was removed from the model if it failed to reach significance (*P* > 0.05). We computed the saddle point using partial derivatives. We examined interaction effects between 25-OHD levels and age, sex and season of blood withdrawal, respectively. All models were adjusted for age, age^2^, sex, education, season, smoking and BMI. In model 1b, we additionally adjusted for osteoporosis status, in model 1c, we additionally adjusted for vitamin D supplementation and in model 1d, we additionally adjusted for physical activity levels. In addition, in sensitivity analyses, we examined whether the association between 25-OHD levels and grip strength changed when (i) excluding participants with neurological diseases, and (ii) when excluding participants with high leverage observations (i.e. individuals with values of predictor variables far off from other observations, which we defined as a hat value greater than 3 times the average). We *z*-standardized all continuous variables to enable comparison of effect sizes. Statistical inferences were made at a two tailed *P* < 0.05. The distribution of residuals was visually inspected to check model diagnostics. All statistical analyses were performed using R (version 4.0.3, The R Foundation, Indianapolis, IN, USA).

### Data availability

The Rhineland Study’s dataset is not publicly available because of data protection regulations. Access to data can be provided to scientists in accordance with the Rhineland Study’s Data Use and Access Policy. Requests for further information or to access the Rhineland Study’s dataset should be directed to RS-DUAC@dzne.de.

## Results

### Demographics

The overall and age-stratified characteristics of the analytical sample are presented in Table 1 and Supplementary Table 1 (see section on supplementary materials given at the end of this article). In total, 1427 women (55.4%) were included. Participants’ age ranged from 30 to 94 years (54.3 ± 14.2 years). In comparison to excluded participants, those who were included were more often male, younger and more physically active and had a higher education status. They suffered less often from neurological diseases and osteoporosis and were less often prescribed cholecalciferol (Table 1).

**Table 1:** Sample demographics of included vs excluded participants of the total sample (*n* = 2999).

	Excluded participants (*n* = 423)	Included participants (*n* = 2576)	Individuals with vitamin D supplementation data (*n* = 2341)	*P*-value^a^
Age (years), mean (s.d.)	60.4 (14.6)	54.3 (14.2)	54.4 (14.0)	<0.001
30–39, *n* (%)	43 (10.2)	477 (18.5)	421 (18.0)	(reference)
40–49, *n* (%)	57 (13.5)	520 (20.2)	479 (20.5)	0.396
50–59, *n* (%)	118 (27.9)	662 (25.7)	596 (25.5)	<0.001
60–69, *n* (%)	61 (14.4)	495 (19.2)	465 (19.9)	0.150
70+, *n* (%)	144 (34.0)	422 (16.4)	380 (16.2)	<0.001
Sex (women), *n* (%)	269 (63.6)	1427 (55.4)	1311 (56.0)	<0.001
Body mass index (kg/m^2^), mean (s.d.)	26.11 (4.68)	25.75 (4.47)	25.71 (4.50)	0.256
Smoking (yes), *n* (%)	46 (11.1)	355 (13.8)	320 (13.7)	0.578
25-hydroxyvitamin D (nmol/L), mean (s.d.)	56.76 (27.98)	55.07 (27.51)	55.53 (28.02)	0.880
Vitamin D categories, *n* (%)				
<30 nmol/L	72 (17.1)	451 (17.5)	408 (17.4)	(reference)
30–<50 nmol/L	107 (25.4)	658 (25.5)	585 (25.0)	0.976
50–≤125 nmol/L	232 (55.1)	1427 (55.4)	1308 (55.9)	0.571
>125 nmol/L	10 (2.4)	40 (1.6)	40 (1.7)	0.601
Vitamin D supplementation status (yes), *n* (%)	136 (35.9)	678 (29.0)	678 (29.0)	0.186
Regular cholecalciferol intake (yes), *n* (%)	75 (18.9)	293 (11.9)	293 (12.7)	0.045
Other vitamin D supplementation (yes), *n* (%)	101 (25.9)	546 (22.8)	546 (23.6)	0.602
Osteoporosis (yes), *n* (%)	48 (11.5)	115 (4.5)	106 (4.6)	0.006
Neurological disease (yes), *n* (%)	23 (5.4)	62 (2.4)	57 (2.4)	0.011
Education ISCED11, *n* (%)				
Low	20 (5.1)	41 (1.6)	35 (1.5)	(reference)
Middle	196 (49.9)	1121 (43.5)	1024 (43.7)	0.006
High	177 (45.0)	1414 (54.9)	1282 (54.8)	0.001
Season of blood withdrawal, *n* (%)				
Spring	89 (21.0)	537 (20.8)	503 (21.5)	(reference)
Summer	95 (22.5)	746 (29.0)	679 (29.0)	0.093
Autumn	157 (37.1)	764 (29.7)	661 (28.2)	0.133
Winter	82 (19.4)	529 (20.5)	498 (21.3)	0.688
Fracture in past 4 weeks (yes), *n* (%)	0 (0.0)	0 (0.0)	0 (0.0)	0.999
Handedness, *n* (%)				
Right	212 (92.6)	2368 (91.9)	2153 (92.0)	(reference)
Left	14 (6.1)	137 (5.3)	126 (5.4)	0.475
Ambidextrous	3 (1.3)	71 (2.8)	62 (2.6)	0.259
Maximum grip strength dominant hand (kg), mean (s.d.)	33.32 (10.52)	36.68 (11.54)	36.56 (11.53)	0.943
Energy expenditure in metabolic equivalent hours, mean (s.d.)	33.63 (1.68)	33.97 (1.43)	33.99 (1.42)	0.005

^a^Differences between included and excluded participants were assessed with logistic regression adjusted for age and sex (group differences for the variables age and sex were only adjusted for the other variable, respectively).

### Association between circulating 25-OHD levels and maximum grip strength of the dominant hand

**Figure 2:**
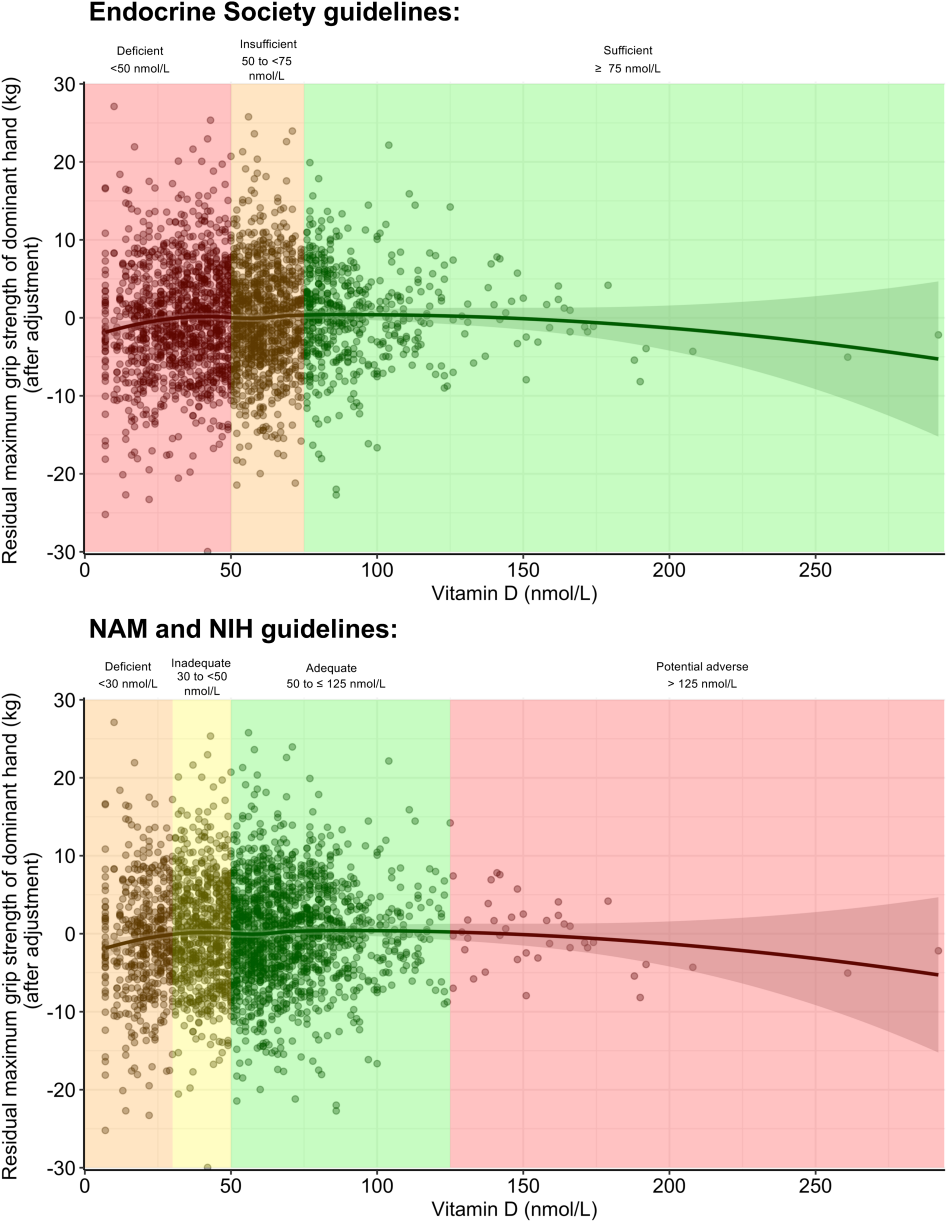
Association between continuous circulating 25-hydroxyvitamin D on maximum grip strength of the dominant hand across vitamin D categories based on the Endocrine Society, National Academy of Medicine (NAM) and National Institutes of Health (NIH) guidelines after adjustment for covariates. Here, we depict the association of circulating 25-hydroxyvitamin and the residuals of the maximum grip strength of the dominant hand with a loess function, after adjusting maximum grip strength of the dominant hand for age, age^2^, sex, education, smoking, BMI and season.

**Figure 3:**
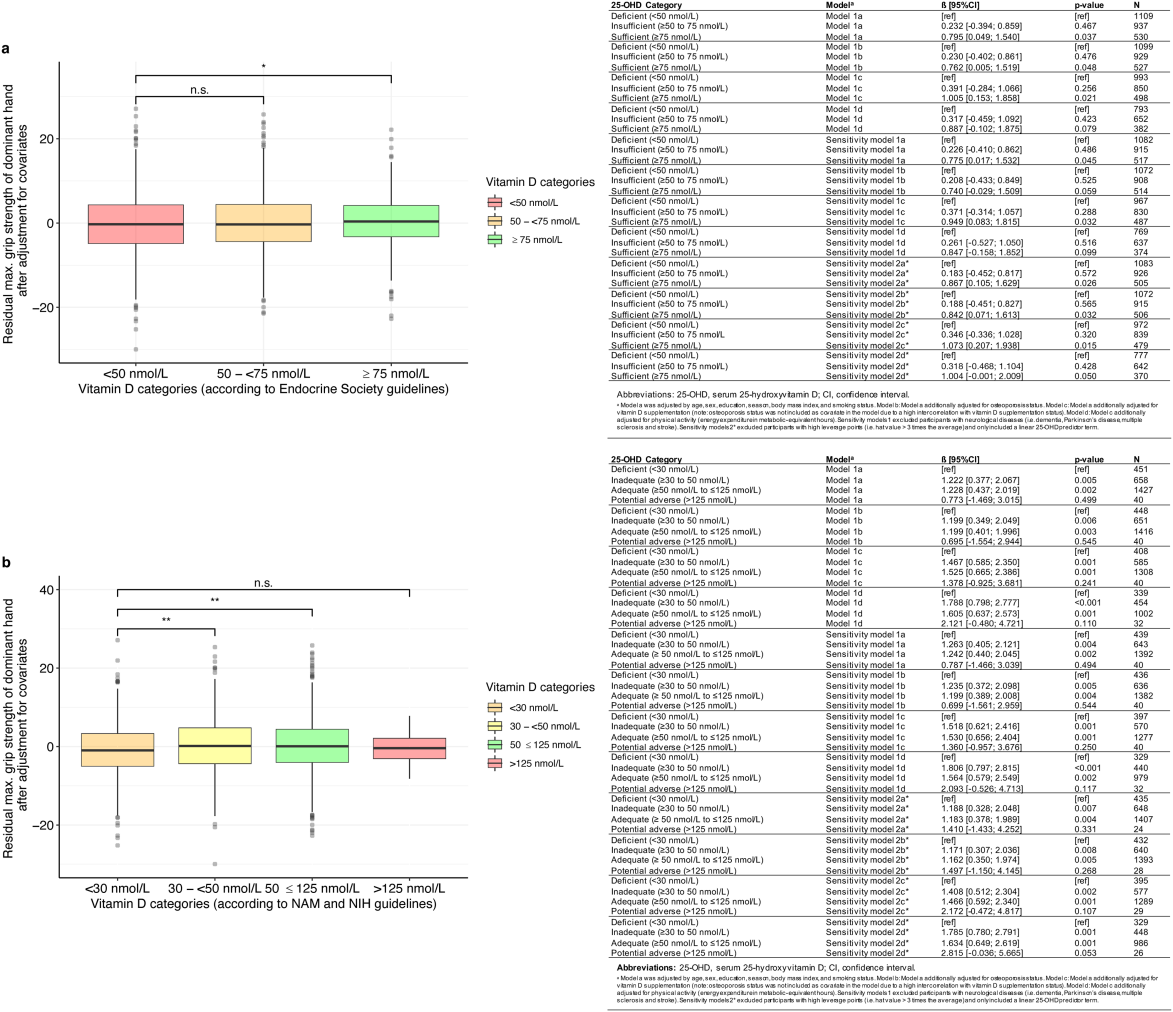
Effects of 25-hydroxyvitamin D (25-OHD) on maximum grip strength of the dominant hand across vitamin D categories after adjustment for covariates. Here, we compare the effects of 25-OHD categories on the residuals of the maximum grip strength of the dominant hand (reference group: deficient) after adjustment for age, age^2^, sex, education, smoking, BMI and season across (A) Endocrine Society categories; (B) National Academy of Medicine (NAM, formerly called Institute of Medicine) and National Institute of Health (NIH) categories.

First, we examined the effects of 25-OHD levels on maximum grip strength of the dominant hand across vitamin D categories (Fig. 2). We used multivariate linear regression models with categorical 25-OHD levels as an independent variable, and we adjusted for age, sex, education, smoking, season and BMI (model 1a). Across the Endocrine Society categories, compared to deficient 25-OHD levels (<50 nmol/L, reference group), maximum grip strength was significantly higher at sufficient (≥75 nmol), but not at insufficient, 25-OHD levels (≥50 to <75 nmol/L) (Fig. 3A). Compared to individuals with deficient 25-OHD levels, the grip strength of individuals with sufficient levels was about 800 g higher on average (model 1a: *ß*_sufficient_ = 0.795; model 1b: *ß*_sufficient_ = 0.762; model 1c: *ß*_sufficient_ = 1.005; model 1d: *ß*_sufficient_ = 0.887). Similarly, across the NAM and NIH categories, maximum grip strength was higher at inadequate (30 to <50 nmol/L) and adequate levels (≥50 to ≤125 nmol/L) compared to deficient 25-OHD levels (<30 nmol/L, reference group; Fig. 3B). Also here we observed that the grip strength of individuals with inadequate and adequate levels was more than 1 kg greater than that of individuals with deficient levels (Fig. 3B). At potential adverse levels (125 nmol/L), we observed vitamin D to be associated with lower grip strength (Fig. 2). Effects at potential adverse levels, however, did not significantly differ from the effects at deficient levels (<30 nmol/L; Fig. 3B). These associations did not change after additional adjustment for osteoporosis status (model 1b), vitamin D supplementation (model 1c) and physical activity levels (model 1d) as well as when excluding participants with neurological diseases (sensitivity models 1a–1d) or high leverage points (sensitivity models 2a–2d; Fig. 3).

**Figure 4:**
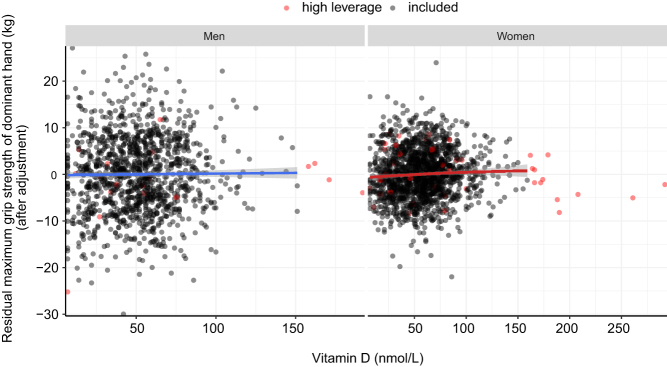
The relation between circulating 25-hydroxyvitamin D and maximum grip strength of the dominant hand stratified by sex after exclusion of participants with high leverage points. Here, we depict the association of circulating 25-hydroxyvitamin and the residuals of the maximum grip strength of the dominant hand, after adjusting the maximum grip strength of the dominant hand for age, age^2^, education, smoking, BMI and season.

Second, we examined the association between continuous 25-OHD levels and maximum grip strength of the dominant hand. We found that standardized 25-OHD levels were associated with a greater maximum handgrip strength of the dominant hand across all models (Table 2). The relation between vitamin D and handgrip strength was curvilinear and most pronounced at low levels of vitamin D (model 1a: *ß*_linear_ = 0.505, 95% CI: 0.179; 0.830, *P* = 0.002; *ß*_quadratic_ = –0.153, 95% CI: –0.269; –0.038, *P* = 0.009). At higher levels, the effects of vitamin D on handgrip strength became progressively weaker and reached the saddle point at 100.4 nmol/L. At even higher levels, 25-OHD levels were associated with a weaker handgrip strength (Fig. 2). After excluding participants with high leverage points (Fig. 4), we found that the relation between vitamin D and handgrip strength became linear (Table 2, sensitivity models 2 and 2*). One s.d. increase in 25-OHD levels was associated with 0.375 kg greater maximum handgrip strength of the dominant hand.

**Table 2:** Main effects of continuous standardized circulating 25-hydroxyvitamin D levels on maximum grip strength of the dominant hand.

Term	Model^a^	*ß* (95% CI)	*P*-value	*n*
25-OHD (linear)	Model 1a	0.505 (0.179; 0.830)	0.002	2576
25-OHD (quadratic)	Model 1a	–0.153 (–0.269; –0.038)	0.009	2576
25-OHD (linear)	Model 1b	0.493 (0.163; 0.823)	0.003	2555
25-OHD (quadratic)	Model 1b	–0.153 (–0.269; –0.037)	0.010	2555
25-OHD (linear)	Model 1c	0.634 (0.257; 1.010)	0.001	2341
25-OHD (quadratic)	Model 1c	–0.162 (–0.283; –0.041)	0.009	2341
25-OHD (linear)	Model 1d	0.629 (0.194; 1.064)	0.005	1827
25-OHD (quadratic)	Model 1d	–0.143 (–0.277; –0.009)	0.036	1827
25-OHD (linear)	Sensitivity model 1a	0.509 (0.178; 0.840)	0.003	2514
25-OHD (quadratic)	Sensitivity model 1a	–0.156 (–0.273; –0.039)	0.009	2514
25-OHD (linear)	Sensitivity model 1b	0.491 (0.156; 0.827)	0.004	2494
25-OHD (quadratic)	Sensitivity model 1b	–0.155 (–0.272; –0.037)	0.010	2494
25-OHD (linear)	Sensitivity model 1c	0.628 (0.245; 1.011)	0.001	2284
25-OHD (quadratic)	Sensitivity model 1c	–0.165 (–0.288; –0.043)	0.008	2284
25-OHD (linear)	Sensitivity model 1d	0.616 (0.172; 1.059)	0.007	1780
25-OHD (quadratic)	Sensitivity model 1d	–0.141 (–0.276; –0.005)	0.042	1780
25-OHD (linear)	Sensitivity model 2a	0.471 (0.156; 0.785)	0.003	2514
25-OHD (quadratic)	Sensitivity model 2a	–0.146 (–0.332; 0.040)	0.125	2514
25-OHD (linear)	Sensitivity model 2b	0.471 (0.150; 0.793)	0.004	2493
25-OHD (quadratic)	Sensitivity model 2b	–0.135 (–0.313; 0.044)	0.138	2493
25-OHD (linear)	Sensitivity model 2c	0.589 (0.225; 0.952)	0.002	2290
25-OHD (quadratic)	Sensitivity model 2c	–0.126 (–0.310; 0.058)	0.180	2290
25-OHD (linear)	Sensitivity model 2d	0.619 (0.193; 1.045)	0.004	1789
25-OHD (quadratic)	Sensitivity model 2d	–0.124 (–0.329; 0.081)	0.236	1789
25-OHD (linear)	Sensitivity model 2a*	0.375 (0.085; 0.665)	0.011	2514
25-OHD (linear)	Sensitivity model 2b*	0.371 (0.078; 0.663)	0.013	2493
25-OHD (linear)	Sensitivity model 2c*	0.494 (0.157; 0.831)	0.004	2290
25-OHD (linear)	Sensitivity model 2d*	0.512 (0.124; 0.900)	0.010	1789

^a^Model a was adjusted by age, sex, education, season, body mass index, and smoking status; Model b: model a additionally adjusted for osteoporosis status. Model c: model a additionally adjusted for vitamin D supplementation status. Note osteoporosis status was not included as covariate in the model due to a high intercorrelation with vitamin D supplementation status. Model d: model c additionally adjusted for physical activity (energy expenditure in metabolic equivalent hours). Sensitivity models 1 excluded participants with neurological diseases (i.e. dementia, Parkinson’s disease, multiple sclerosis and stroke). Sensitivity models 2 excluded participants with high leverage points (i.e. hat value greater than three times the average). Sensitivity models 2* excluded participants with high leverage points (i.e. hat value greater than three times the average) and only included a linear 25-OHD predictor term.

25-OHD, serum 25-hydroxyvitamin D.

### Interactions between circulating 25-OHD levels, age, sex and season

**Figure 5:**
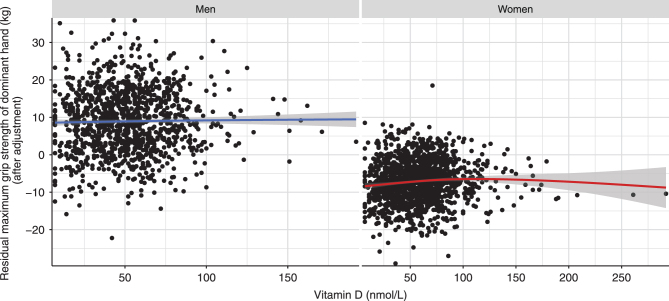
Effects of circulating 25-hydroxyvitamin D levels on the maximum grip strength of the dominant hand stratified by sex. Here, we depict the association of circulating 25-hydroxyvitamin and the residuals of the maximum grip strength of the dominant hand, after adjusting maximum grip strength of the dominant hand for age, age^2^, education, smoking, BMI and season.

Lastly, we assessed whether the effect of vitamin D on maximum grip strength of the dominant hand changed across age, between seasons and differed between men and women. The association between 25-OHD levels and grip strength did not differ between seasons nor between men and women (Table 3, Fig. 5).

**Table 3:** Interaction between continuous circulating 25-hydroxyvitamin D, age, sex and season.

Interaction term	Model^a^	ß (95% CI)	*P*-value	*n*
25-OHD × age				
	Model 1a	–0.309 (–0.594; –0.024)	0.033	2576
	Model 1b	–0.323 (–0.612; –0.034)	0.029	2555
	Model 1c	–0.213 (–0.514; 0.087)	0.164	2341
	Model 1d	–0.159 (–0.499; 0.180)	0.357	1827
	Sensitivity model 1a	–0.321 (–0.609; –0.033)	0.029	2514
	Sensitivity model 1b	–0.342 (–0.634; –0.050)	0.022	2494
	Sensitivity model 1c	–0.222 (–0.526; 0.082)	0.152	2284
	Sensitivity model 1d	–0.187 (–0.531; 0.157)	0.287	1780
	Sensitivity model 2a*	–0.329 (–0.602; –0.056)	0.018	2514
	Sensitivity model 2b*	–0.351 (–0.629; –0.073)	0.013	2493
	Sensitivity model 2c*	–0.240 (–0.527; 0.047)	0.102	2290
	Sensitivity model 2d*	–0.197 (0.526; 0.131)	0.240	1789
25-OHD × sex				
	Model 1a	0.035 (–0.535; 0.606)	0.904	2576
	Model 1b	0.038 (–0.537, 0.613)	0.897	2555
	Model 1c	0.200 (–0.397; 0.797)	0.511	2341
	Model 1d	0.110 (–0.582; 0.802)	0.754	1827
	Sensitivity model 1a	0.037 (–0.543; 0.618)	0.900	2514
	Sensitivity model 1b	0.031 (–0.554; 0.616)	0.918	2494
	Sensitivity model 1c	0.195 (–0.413; 0.802)	0.530	2284
	Sensitivity model 1d	0.143 (–0.564; 0.849)	0.692	1780
	Sensitivity model 2a*	0.014 (–0.539; 0.567)	0.960	2514
	Sensitivity model 2b*	0.001 (–0.558; 0.559)	0.999	2493
	Sensitivity model 2c*	0.177 (–0.399; 0.753)	0.546	2290
	Sensitivity model 2d*	0.057 (–0.604; 0.719)	0.865	1789
25-OHD × season				
Spring	Model 1a	(reference)	(reference)	2576
Summer	Model 1a	0.132 (–0.740; 1.005)	0.766	2576
Autumn	Model 1a	–0.191 (–0.981; 0.599)	0.636	2576
Winter	Model 1a	–0.586 (–1.442; 0.270)	0.180	2576
Spring	Model 1b	(reference)	(reference)	2555
Summer	Model 1b	0.133 (–0.745; 1.011)	0.767	2555
Autumn	Model 1b	–0.227 (–1.021; 0.566)	0.574	2555
Winter	Model 1b	–0.579 (–1.440; 0.281)	0.187	2555
Spring	Model 1c	(reference)	(reference)	2341
Summer	Model 1c	0.147 (–0.763; 1.057)	0.751	2341
Autumn	Model 1c	–0.146 (–0.970; 0.677)	0.728	2341
Winter	Model 1c	–0.531 (–1.411; 0.349)	0.237	2341
Spring	Model 1d	(reference)	(reference)	1827
Summer	Model 1d	0.134 (–0.906; 1.174)	0.800	1827
Autumn	Model 1d	–0.167 (–1.111; 0.778)	0.730	1827
Winter	Model 1d	–0.666 (–1.674; 0.342)	0.195	1827
Spring	Sensitivity model 1a	(reference)	(reference)	2514
Summer	Sensitivity model 1a	0.056 (–0.830; 0.943)	0.901	2514
Autumn	Sensitivity model 1a	–0.221 (–1.022; 0.581)	0.590	2514
Winter	Sensitivity model 1a	–0.662 (–1.531; 0.207)	0.135	2514
Spring	Sensitivity model 1b	(reference)	(reference)	2494
Summer	Sensitivity model 1b	0.040 (–0.852; 0.932)	0.930	2494
Autumn	Sensitivity model 1b	–0.268 (–1.074; 0.538)	0.514	2494
Winter	Sensitivity model 1b	–0.665 (–1.540; 0.209)	0.136	2494
Spring	Sensitivity model 1c	(reference)	(reference)	2284
Summer	Sensitivity model 1c	0.089 (–0.835; 1.013)	0.850	2284
Autumn	Sensitivity model 1c	–0.157 (–0.994; 0.679)	0.713	2284
Winter	Sensitivity model 1c	–0.601 (–1.494; 0.292)	0.187	2284
Spring	Sensitivity model 1d	(reference)	(reference)	1780
Summer	Sensitivity model 1d	0.060 (–0.999; 1.119)	0.911	1780
Autumn	Sensitivity model 1d	–0.195 (–1.157; 0.766)	0.691	1780
Winter	Sensitivity model 1d	–0.744 (–1.766; 0.279)	0.154	1780
Spring	Sensitivity model 2a*	(reference)	(reference)	2514
Summer	Sensitivity model 2a*	0.077 (–0.788; 0.943)	0.861	2514
Autumn	Sensitivity model 2a*	–0.208 (–0.996; 0.581)	0.606	2514
Winter	Sensitivity model 2a*	–0.558 (–1.386; 0.27)	0.186	2514
Spring	Sensitivity model 2b*	(reference)	(reference)	2493
Summer	Sensitivity model 2b*	0.043 (–0.817; 0.902)	0.923	2493
Autumn	Sensitivity model 2b*	–0.266 (–1.047; 0.516)	0.505	2493
Winter	Sensitivity model 2b*	–0.572 (–1.400; 0.257)	0.176	2493
Spring	Sensitivity model 2c*	(reference)	(reference)	2290
Summer	Sensitivity model 2c*	0.035 (–0.852; 0.922)	0.938	2290
Autumn	Sensitivity model 2c*	–0.196 (–1.003; 0.611)	0.634	2290
Winter	Sensitivity model 2c*	–0.528 (–1.374; 0.318)	0.221	2290
Spring	Sensitivity model 2d*	(reference)	(reference)	1789
Summer	Sensitivity model 2d*	0.017 (–0.996; 1.029)	0.974	1789
Autumn	Sensitivity model 2d*	–0.161 (–1.085; 0.764)	0.734	1789
Winter	Sensitivity model 2d*	–0.545 (–1.502; 0.412)	0.264	1789

^a^Model a was adjusted by age, sex, education, season, body mass index and smoking status. Model b: model a additionally adjusted for osteoporosis status. Model c: model a additionally adjusted for vitamin D supplementation status. Note osteoporosis status was not included as covariate in the model due to a high intercorrelation with vitamin D supplementation status. Model d: model c additionally adjusted for physical activity (energy expenditure in metabolic-equivalent hours). Sensitivity models 1 excluded participants with neurological diseases (i.e. dementia, Parkinson’s disease, multiple sclerosis and stroke). Sensitivity models 2* excluded participants with high leverage points (i.e. hat value greater than three times the average) and only included a linear 25-OHD predictor term.

25-OHD, serum 25-hydroxyvitamin D.

**Figure 6:**
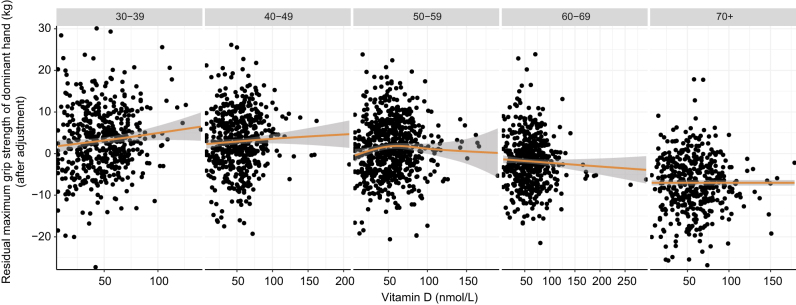
Effects of circulating 25-hydroxyvitamin D levels on maximum grip strength of the dominant hand stratified by age. Here, we depict the association of circulating 25-hydroxyvitamin and the residuals of the maximum grip strength of the dominant hand, after adjusting maximum grip strength of the dominant hand for sex, education, smoking, BMI and season.

**Figure 7:**
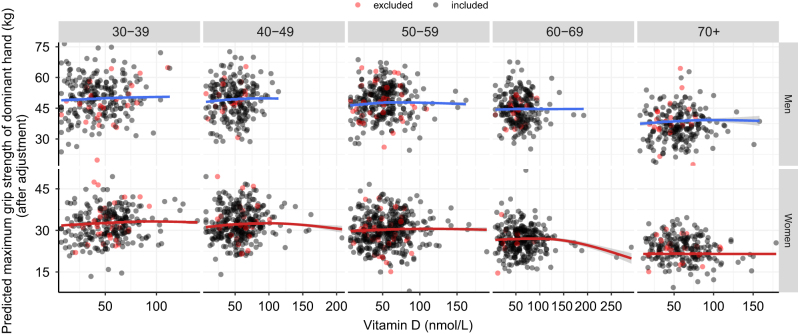
The relation between circulating 25-hydroxyvitamin D and maximum grip strength of the dominant hand stratified by age and sex after exclusion of participants with missing data on vitamin D supplementation. Regression lines were adjusted for age, age^2^, sex, education, smoking, BMI, season and vitamin D supplementation.

However, age had a significant moderating effect on this association, after adjusting for sex, education, smoking, season and BMI (model 1a: *ß*_25OHDxAge_ = –0.309, 95% CI: –0.594; –0.024, *P* = 0.033), as well when additionally adjusting for osteoporosis status (model 1b: *ß*_25OHDxAge_ = –0.323, 95% CI: –0.612; –0.034, *P* = 0.029) but not when additionally adjusting for vitamin D supplementation (model 1c: *ß*_25OHDxAge_ = –0.213, 95% CI: –0.514; 0.087, *P* = 0.164) and physical activity levels (model 1d: *ß*_25OHDxAge_ = –0.159, 95% CI: –0.499; 0.180, *P* = 0.357). We observed that the effect of vitamin D on grip strength was weaker in older adults compared to younger adults, both in men and in women (Fig. 6). Particularly in older women, we observed extremely high levels of vitamin D to be associated with lower grip strength. When adjusting for vitamin D supplementation, several older individuals with high leverage points were excluded due to missing data on vitamin D supplementation (Fig. 7).

## Discussion

We aimed to examine the relation between circulating 25-OHD levels and handgrip strength across adult life span in a large population-based cohort study. In our cross-sectional sample of 2576 individuals aged 30–94 years, we found a robust association between circulating vitamin D levels and maximum grip strength of the dominant hand after adjustment for age, sex, education, season, smoking, BMI, physical activity, osteoporosis status and vitamin D supplementation. Our findings highlight the importance of adequate vitamin D levels for the maintenance of muscle function in adults across a wide age range.

Thus far, findings from observational studies examining the relation between vitamin D levels and grip strength have been contradictory (13, 15, 17, 19, 20). While some studies reported higher vitamin D levels to be associated with greater upper arm and grip strength (13, 15), others could not replicate these findings (29, 30). The heterogeneity of these findings may reflect different study designs and populations: particularly, differences in skin pigmentation, latitude, dietary patterns, lifestyle factors as well as genetic polymorphisms should be taken into account when comparing findings of studies across different regions and ethnicities (31, 32, 33). In addition, varying vitamin D thresholds have been used to cluster participants and to define vitamin D deficiency across studies with an ongoing controversy about recommended vitamin D targets (34). This hampers comparisons of the findings of different studies and may lead to biased conclusions (35).

Thus, to allow better comparison to previous findings, here we report results based on the Endocrine Society’s, the NAM and the NIH’s classification guidelines for circulating 25-OHD levels (36, 37, 38). Across NAM and NIH categories, we found that individuals with deficient 25-OHD levels (<30 nmol/L) show lower grip strength than individuals with inadequate (30 to <50 nmol/L) or adequate levels (≥50 to ≤125 nmol/L). However, no differences in the effects of 25-OHD levels on grip strength between the deficient (<30 nmol/L) and the potential adverse (>125 nmol/L) categories were observed. Across the Endocrine Society’s categories, we only found differences in the effects on grip strength between individuals having deficient 25-OHD levels (<50 nmol/L) and individuals having sufficient levels (≥75 nmol) but not between individuals having deficient 25-OHD levels and individuals having insufficient levels (≥50 to <75 nmol/L). Individuals with sufficient 25-OHD levels had, on average, more than 1 kg higher grip strength than individuals with deficient levels. In comparison, grip strength across the life span trajectory has been observed to follow a curvilinear trend: with the highest values in early adulthood (30–39 years) and loss of grip strength by about 0.3 kg and 0.6 kg per decade in middle (40–49 years) and late (50–59 years) adulthood, respectively and a rapid decline thereafter (39, 40).

Next to dividing 25-OHD levels into categories, we also examined the association between vitamin D and grip strength on a continuous scale. We could replicate findings from a previous cohort study, which examined the effects of continuous vitamin D levels in 419 healthy men and women aged 20–76 years and reported a positive effect on upper arm strength (13). Specifically, we found that the effect of vitamin D on grip strength was strongest at low levels of 25-OHD (<50 nmol/L) and weakened at higher levels. We observed the maximum effect at around 100 nmol/L. At even higher levels, we observed a strong negative effect of 25-OHD levels on grip strength across all ages. Taken together, in line with NAM and NIH recommendations (36, 38), our findings suggest a dose-response relationship between vitamin D levels and grip strength with an optimum around 50–100 nmol/L and an increased risk of adverse effects at excess levels of circulating 25-OHD levels above 125 nmol/L. Moreover, our findings highlight that examining the effects of 25-OHD across narrow categories, such as proposed by the NAM and NIH guidelines, or examining the effects of 25-OHD levels on a continuous scale may provide valuable insights such as the potential adverse effects at excess 25-OHD levels that may be hidden when using broad cutoffs. Nonetheless, it should be noted that we detected potential adverse vitamin D levels only in a few participants. Further studies are warranted to study the association between extreme vitamin D levels and grip strength in greater detail.

While previous studies largely focused on older adults, our study examined the association between vitamin D levels and grip strength in adults across a wide age range. This allowed us to examine whether the effect of vitamin D is modified by age. Surprisingly, we observed a weaker effect of vitamin D on grip strength in older adults compared to younger adults. A systematic review of randomized controlled trials found that overall muscle strength of older adults could profit more from vitamin D supplementation than that of younger adults (41). Compared to other cohort studies in Germany (35, 42), a substantially higher percentage of our participants reported to take vitamin D supplementation (29.0% vs 2.81% men and 3.8% women of the German National Health Interview and Examination Survey (GNHIES) 1998 (42)) and a substantially lower percentage of our participants were showing a vitamin D deficiency (43.0% < 50 nmol/L vs 56.8% < 50 nmol/L in the GNHIES 1998 (42) and ~62.1% <50 nmol/L in the Studie zur Gesundheit Erwachsener in Deutschland (DEGS1) (43)). This is in line with a recent report observing a drastic increase in prescribed cholecalciferol in recent years (44). In our sample, we observed that compared to younger individuals, a greater proportion of older adults had been regularly taking prescribed cholecalciferol and had higher vitamin D levels. Indeed, we found that after accounting for vitamin D supplementation, the relation between vitamin D levels and grip strength did not change with age.

In addition, we aimed to examine whether the effects of circulating vitamin D levels on grip strength varied between seasons and differed between men and women. Little is known about seasonal variations of muscle function in relation to vitamin D levels. Bird and colleagues (2013) found seasonal variations in ankle strength and serum vitamin D levels in community-dwelling older adults. Similarly, Milani and colleagues (2021) reported season-dependent effects of vitamin D on physical fitness performance in male adolescents. To the best of our knowledge, no study to date has examined the modifying effects of season or sex on the association between vitamin D and grip strength in adults across a wide age range. In our study, we observed a modifying effect neither of season nor of sex. Several observational studies noticed an association between vitamin D levels and grip strength exclusively in healthy middle-aged men (15) and older men (17) or stronger associations in older men than in older women (20). However, other studies observed effects both in older men and in women (19, 45). A recent narrative review points to potential sex differences in the synthesis and metabolism of vitamin D, which may lead to differences in the effects of vitamin D on grip strength between men and women (46). Further research is needed to study factors that could modulate the effects of vitamin D on muscle strength.

A number of limitations of our study should be noted. First, this study examined cross-sectional associations between vitamin D levels and grip strength using baseline data of a large cohort study. Therefore, the longitudinal relationship between circulating vitamin D levels and grip strength could not be explored, and a causal relationship cannot be inferred. Second, given the relatively high education levels and the high prevalence of vitamin D supplementation across all age groups in our sample, it cannot be ruled out that our findings may have been partly influenced by selection bias. Third, our findings in predominantly white individuals from European descent may not be generalizable to other ethnicities. Fourth, while grip strength has been found to be a reliable proxy of muscle strength (4, 5), several studies highlight the usefulness of measuring muscle strength of multiple muscle groups to achieve more precise estimates of overall muscle function (47, 48).

To conclude, here we present a detailed characterization of the relation between circulating 25-OHD levels and muscle strength in adults aged 30–94 years. We observed a robust association between vitamin D levels and grip strength, which was most pronounced at deficient levels and dropped at potential adverse levels. Our findings suggest that optimizing vitamin D levels could be an easily actionable and inexpensive strategy for improving muscle function and protecting against sarcopenia in adults across a wide age range. Nonetheless, vitamin D supplementation should be closely monitored to avoid overdosing and potential detrimental effects.

## Supplementary Material

Supplementary Table 1. Age-stratified demographics.

## Declaration of interest

The authors report no competing interests.

## Funding

The Rhineland Study at the DZNE is predominantly funded by the Federal Ministry of Education and Research (BMBF) and the Ministry of Culture and Science of the German State of North Rhine-Westphalia. Dr Aziz is partly supported by an Alzheimer's Association Research Grant (award number: AARG-19-616534) and an European Research Council Starting Grant (number: 101041677).

## Author contribution statement

Fabienne AU Fox: Conceptualization, Methodology, Formal Analysis, Writing – Original Draft Preparation, Visualization, Supervision; Lennart Koch: Methodology, Formal Analysis, Writing – Original Draft Preparation, Visualization; Monique MB Breteler: Conceptualization, Methodology, Resources, Writing – Reviewing and Editing, Data Curation, Funding Acquisition; N Ahmad Aziz: Conceptualization, Methodology, Supervision, Writing – Reviewing and Editing.
