# Supplementary material for: 25-hydroxyvitamin D level is associated with greater grip strength across adult life span: a population-based cohort study
Source: Endocr Connect. 2023 Mar 28;12(4):e220501. doi: 10.1530/EC-22-0501 (PMC10083672; doi:10.1530/EC-22-0501)
Supplement: Supplementary Table 1. Age-stratified demographics. [file supplementary_table_1.pdf]

Supplementary Materials

Supplementary Table 1. Age-stratified demographics.

	30-39 years	40-49 years	50-59 years	60-69 years	70+ years
Sex (women), n (%)	252 (52.8)	306 (58.8)	384 (58.0)	274 (55.4)	211 (50.0)
Body-mass index (kg/m^2), mean (SD)	24.56 (4.20)	25.78 (5.10)	25.90 (4.41)	26.36 (4.42)	26.12 (3.85)
Smoking (yes), n (%)	83 (17.4)	64 (12.3)	115 (17.4)	71 (14.3)	22 (5.2)
25-hydroxyvitamin D (nmol/L), mean (SD)	48.87 (23.14)	53.42 (25.55)	54.28 (27.20)	60.79 (32.41)	58.65 (26.94)
Vitamin D categories, n (%)					
<30 nmol/L	99 (20.8)	90 (17.3)	131 (19.8)	75 (15.2)	56 (13.3)
30 - <50 nmol/L	145 (30.4)	134 (25.8)	169 (25.5)	107 (21.6)	103 (24.4)
50 - ≤125 nmol/L	231 (48.4)	290 (55.8)	351 (53.0)	301 (60.8)	254 (60.2)
>125 nmol/L	2 (0.4)	6 (1.2)	11 (1.7)	12 (2.4)	9 (2.1)
Vitamin D supplementation status (yes), n (%)	97 (23.0)	121 (25.3)	159 (26.7)	157 (33.8)	144 (37.9)
Regular cholecalciferol intake (yes), n (%)	19 (4.2)	50 (10.0)	65 (10.3)	78 (16.5)	81 (20.4)
Other vitamin D supplementation (yes), n (%)	90 (20.7)	99 (20.5)	137 (22.3)	118 (25.1)	102 (26.2)
Osteoporosis (yes), n (%)	2 (0.4)	4 (0.8)	16 (2.4)	34 (6.9)	59 (14.4)
Neurological disease (yes), n (%)	8 (1.7)	8 (1.5)	10 (1.5)	14 (2.8)	22 (5.2)
Education ISCED11, n (%)					
low	3 (0.6)	4 (0.8)	7 (1.1)	13 (2.6)	14 (3.3)
middle	158 (33.1)	181 (34.8)	291 (44.0)	248 (50.1)	243 (57.6)
high	316 (66.2)	335 (64.4)	364 (55.0)	234 (47.3)	165 (39.1)
Season of blood withdrawal, n (%)					
spring	102 (21.4)	100 (19.2)	139 (21.0)	111 (22.4)	85 (20.1)
summer	137 (28.7)	148 (28.5)	186 (28.1)	141 (28.5)	134 (31.8)
autumn	137 (28.7)	157 (30.2)	191 (28.9)	149 (30.1)	130 (30.8)
winter	101 (21.2)	115 (22.1)	146 (22.1)	94 (19.0)	73 (17.3)
Fracture in past 4 weeks (yes), n (%)	0 (0.0)	0 (0.0)	0 (0.0)	0 (0.0)	0 (0.0)
Handedness, n (%)					
right	428 (89.7)	482 (92.7)	614 (92.7)	460 (92.9)	384 (91.0)
left	36 (7.5)	26 (5.0)	35 (5.3)	17 (3.4)	23 (5.5)
ambidextrous	13 (2.7)	12 (2.3)	13 (2.0)	18 (3.6)	15 (3.6)
Max. grip strength dominant hand (kg), mean (SD)	40.53 (11.99)	39.27 (10.86)	37.63 (11.42)	34.64 (10.90)	30.04 (9.34)
Energy expenditure in metabolic-equivalent (MET) hours, mean (SD)	34.02 (1.50)	34.17 (1.37)	34.14 (1.47)	33.89 (1.30)	33.56 (1.37)
